# Student-generated learning materials as active learning: a qualitative study of midwifery students’ experiences

**DOI:** 10.1186/s12909-026-09749-9

**Published:** 2026-07-01

**Authors:** Seda Serhatlıoğlu

**Affiliations:** Department of Midwifery, Faculty of Health Sciences, University of Antalya Bilim, Çıplaklı, Akdeniz Blv. No:290/A, Döşemealtı, Antalya, 07190 Türkiye

**Keywords:** Active learning, Health education, Learning materials, Midwifery, Qualitative research

## Abstract

**Background/Objectives:**

Student generated learning materials are increasingly used in health education to support active learning. However, evidence specifically addressing midwifery students’ perceptions of this process in course-based educational contexts remains limited. This exploratory qualitative study aimed to examine midwifery students’ written reflections on the process of generating learning materials within the “Normal Birth and Postpartum Period” course.

**Methods:**

The study was conducted with third-year midwifery students at a foundation university in southern Türkiye. Of 39 eligible students, 26 provided usable structured open-ended written reflection data. Students generated learning materials related to course topics over a 10-week period and presented them during an end of term exhibition. Data were collected after course assessment and formal grading procedures had been completed. The written reflections were analyzed using reflexive thematic analysis. The study followed the COREQ checklist, and ethical approval was obtained.

**Results:**

Analysis revealed four main themes with thirteen sub-themes: individual learning and academic development, emotional and personal experiences, difficulties and obstacles, and instructional direction and application experience. Students perceived the process as supporting engagement with course content, visual learning, self-confidence, and professional awareness. However, students also reported stress, time management difficulties, material-related limitations, uncertainty in topic or material selection, and limited satisfaction in some cases.

**Conclusion:**

Since this exploratory qualitative study was based on written reflections from a single cohort, the findings should be interpreted as students’ self-reported perceptions rather than evidence of objective learning outcomes or pedagogical effectiveness. Further studies using mixed-methods designs, objective learning measures, and diverse educational settings are needed.

**Supplementary Information:**

The online version contains supplementary material available at https://doi.org/10.1186/s12909-026-09749-9.

## Background

Midwifery, as a professional health discipline that combines scientific knowledge and art, is an indispensable part of modern health systems in women’s health and childbirth services [1, 2]. International organizations emphasize that strengthening midwifery education is an important component of improving the quality of maternal and newborn care [1–4]. Improving the quality of midwifery education will be possible through the rapid and accurate transfer of theoretical knowledge into clinical practice. Therefore, midwifery students must be equipped not only with theoretical knowledge but also with multifaceted competencies such as psychomotor skills, communication, problem solving, and clinical decision-making [2, 3, 5].

Student-centered and active learning approaches are increasingly recommended in health professions education to support students’ engagement with theoretical knowledge and professional development [3, 5]. In this regard, strategies such as simulations, scenario-based instruction, modeling, and student-generated learning materials (SGLM) support both the reinforcement of theoretical knowledge and the formation of professional identity [6–8]. According to Hattie’s landmark meta-analyses on visible learning, student-generated teaching and learning interventions yield robust educational effect sizes, demonstrating that when students actively construct instructional artifacts, their conceptual mastery increases significantly [9]. Specifically, SGLM tasks engage learners in structuring, visualizing, and applying knowledge, while fostering autonomy and self-regulated learning [10, 11]. Consistent with Kolb’s experiential learning theory, such participation support the internalization of knowledge and its transfer to practice [12, 13]. In Kolb’s cycle, preparing learning materials represented a concrete experience; students’ written reflections corresponded to reflective observation; transforming theoretical knowledge into visual, tactile, or digital materials reflected abstract conceptualization; and presenting or applying these materials within the course context represented active experimentation. The process of SGLM is also important in terms of making learning permanent and appealing to different learning styles [10, 11, 14].

Indeed, the literature shows that practices in which students contribute to the educational process through art, creativity, and handmade items support learning [15]. Particularly in Obstetrics and Gynecology education, where abstract concepts are prevalent, integrating handmade models into education facilitates conceptual learning and provides a practical, low-cost alternative [14, 16, 17]. However, the limited accessibility of ready-made simulators makes handmade materials valuable from both pedagogical and practical perspectives. At the same time, these benefits should be balanced against the risk of cognitive load. According to cognitive load theory, learners have limited working memory capacity, and complex tasks may overload cognitive processing when students are required to simultaneously understand content, design representations, produce materials, and prepare explanations [18]. Therefore, SGLM tasks require clear guidance, adequate scaffolding, and alignment with students’ prior knowledge and course objectives [19].

SGLM and active learning approaches have been widely examined in health professions education, and recent reviews indicate that student-made models and creative projects are associated with perceived learning benefits, engagement, creativity, and opportunities for learner-centered participation [11, 14]. However, while systematic reviews and broader health professions literature have evaluated student-generated content extensively, qualitative evidence exploring the deep experiential nuances of this process specifically within undergraduate midwifery curricula remains sparse. In this context, active and experiential approaches are particularly relevant for birth and postpartum care courses that combine theoretical and practice-oriented learning. This study aims to address this gap by exploring midwifery students’ experiences with SGLM tasks as part of their coursework in Türkiye. The findings may also offer practical insights for educators seeking to integrate SGLM into course-based activities, particularly in educational settings where access to advanced simulation resources is limited.

## Methods

### Study design

This exploratory qualitative descriptive study analyzed students’ written reflections using thematic analysis. The study was reported in accordance with the COREQ qualitative reporting guidelines (see Supporting Table 1).

### Sample and setting

The study was conducted during the fall semester of the 2024–2025 academic year at the Midwifery Department of a foundation university in southern Türkiye. The study group consisted of third-year midwifery students (*n* = 39) who were asked to SGLM as part of the “Normal Birth and Postpartum Period” course. The remaining 13 students were classified as non-participants because they either left the open-ended written reflection form completely blank, did not return the form, or provided responses that were too incomplete to be included in the qualitative analysis. No student explicitly refused to participate or formally withdrew from the study after being invited. Participation in the qualitative component was voluntary and non-participation had no effect on course grades, jury evaluation, academic assessment, or the instructor–student relationship.

### Evaluation of course content and learning materials

In Türkiye, undergraduate midwifery education is delivered at the bachelor’s degree level and includes both theoretical coursework and practice learning experiences. The “Normal Birth and Postpartum Period” course is a core professional course because it addresses key midwifery competencies related to the physiology and stages of birth, cardinal movements of the fetus, pelvic dynamics, intrapartum care, postpartum care, breastfeeding, and maternal-newborn health [20]. Because the course includes anatomical, physiological, clinical, and educational content related to birth and postpartum care, it provides a suitable context for SGLM activities that require students to transform theoretical knowledge into visual, tactile, and educational materials.

Students were asked to generate interactive, original, and learning materials based on one of these topics and were given 10 weeks to do so. The type of materials was left to the students’ discretion (e.g., poster, model, digital content, game, etc.). Students were encouraged to use accessible, recyclable, locally available, or low-cost materials, and no specific commercial or high-cost product was required. Material cost was not used as an evaluation criterion. Topic selection was student-led; however, the course instructor provided guidance to ensure alignment with course objectives, prevent excessive topic overlap, and maintain diversity among the generated materials. When more than one student or group selected a similar topic, they were encouraged to differentiate their materials in terms of focus, target audience, visual format, or educational purpose.

**Fig. 1:**
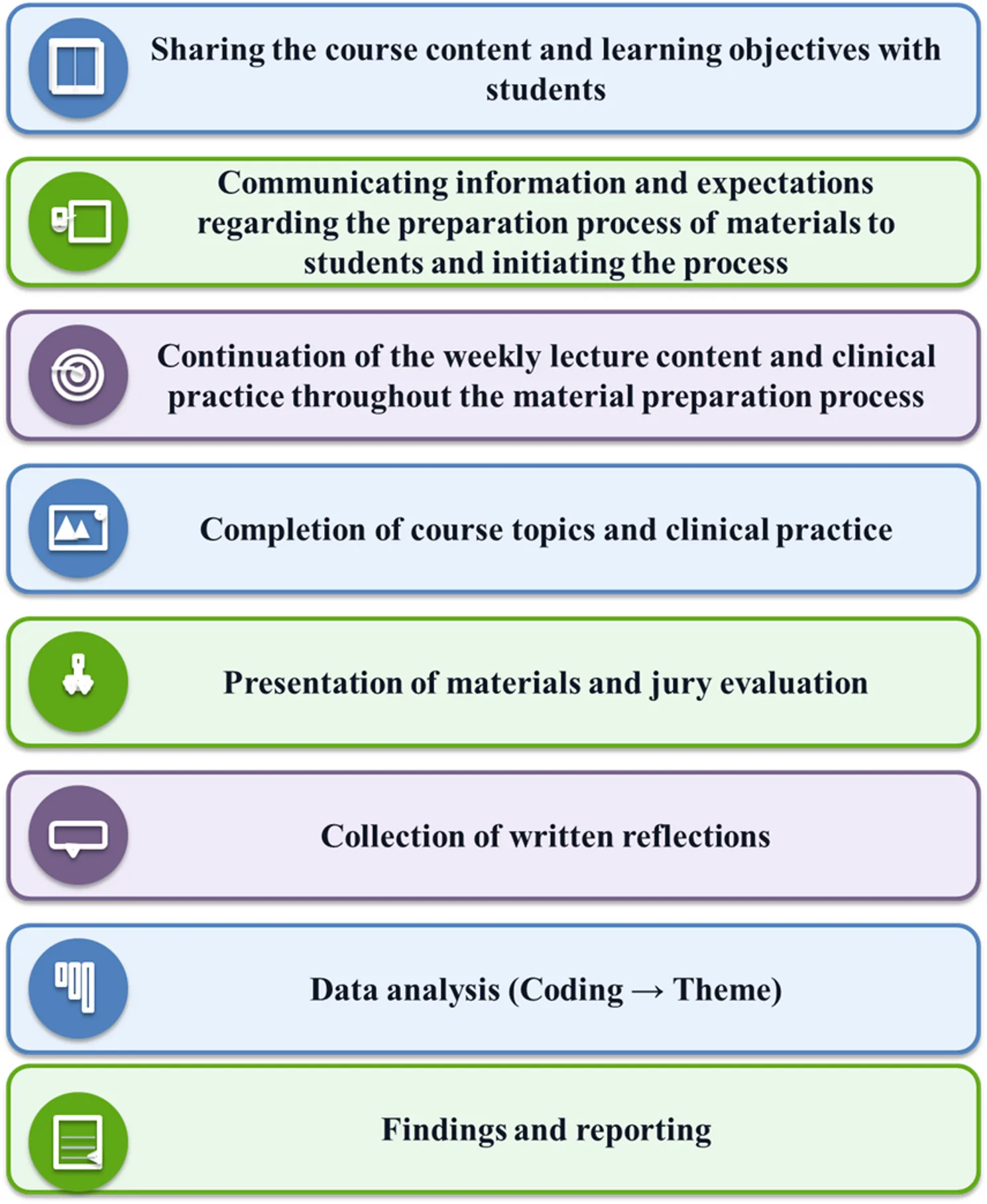
Research flow chart

SGLM were evaluated in an organized exhibition environment by an independent jury (2 midwives, 3 obstetric nurses) to support the reliability of the process, based on criteria such as scientific accuracy, content appropriateness, visual design, creativity and originality, pedagogical impact, and educational suitability. During the evaluation, students were asked to explain the material they prepared within five minutes so that their learning potential and presentation skills could also be observed. Each material was scored between 1 (inadequate) and 5 (excellent) according to the given criteria. The SGLM activity was not included in course grading and did not contribute to students’ exam scores or final course grades. The materials were evaluated only by a jury using a rubric for exhibition purposes and these jury evaluation scores were not used as research data. The jury evaluation was used only to ensure the reliability of the process (Fig. 1).

Kolb’s experiential learning cycle was used as a sensitizing framework, while coding and theme development were conducted inductively based on students’ written reflections [12]. SGLM were classified into two groups: physical models (breast, placenta, pelvic bones, etc.) and artistic projects (paintings, handicrafts, knitting, etc.). Since some materials addressed more than one topic area, topic categories were not treated as mutually exclusive. Overall, the SGLM mainly focused on the birth process, anatomical structures, and the postpartum period. To improve transparency regarding the scope and diversity of the generated materials, selected examples are presented in Supplementary Fig. 1.The most frequently represented topic was the stages of birth (8/30 = 26.7%). Nearly all of the SGLM (22/26 = 84.6%) were of the “hands-on model” type. The materials used in the material process varied, including wood, metal, knitting yarns, felt, ceramics, clay, cardboard, etc. (see Supplementary Table 2).

### Data collection and tools

Data were collected through structured, open-ended written reflection forms rather than oral interviews. This methodological choice was based on operational and ethical considerations: it allowed participating students to reflect within the same educational context, reduced the potential pressure associated with face to face researcher–student interviews, and supported anonymity during data collection. Although written reflections lack real-time probing, non-verbal cues, and the conversational depth of in-depth interviews, this format provided a uniform and systematic framework for documenting students’ immediate course-based experiences.

Written data was collected anonymously from 26 students who participated in the material preparation process and volunteered to participate in the study, using a form consisting of open-ended questions (Table 1). The form included six open-ended questions aimed at revealing the students’ experiences, perceptions, difficulties they encountered, and what this process contributed to them. The questions were developed by the researchers based on a literature review and expert opinion [11, 12]. The written reflection form was piloted with five fourth-year midwifery students who were not included in the main study. Based on their feedback, minor wording revisions were made to improve clarity, with no substantial changes to question content. The researcher gave the students code numbers and structured interview forms. Students were given codes from 1 to 26 (Student 1, Student 2, Student 25, Student 26). Students who did not participate were not given any alternative assignment or additional task.

**Table 1:** Questions in the data collection form

Questions
1-How did you find the process of SGLM in general?
2-How did the process of SGLM contribute to your learning?
3-What difficulties did you encounter while generating the materials?
4-Do you think the material you generated is usable? Why?
5-What are your suggestions regarding the SGLM process?
6-Would you like to share any additional comments about the process in general?

### Data analysis

The qualitative data obtained from written responses were evaluated using Braun and Clarke’s (2022) six-step reflexive thematic analysis method. These stages are: (1) Familiarization with the data, (2) Generating initial codes, (3) Generating initial themes, (4) Reviewing potential themes, (5) Defining and naming themes, and (6) Producing the report [21]. The coding process was conducted by two experts; consensus was achieved. Because the study used a reflexive thematic analysis approach, a statistical intercoder reliability coefficient, such as Cohen’s kappa, was not calculated. Instead, analytic transparency was supported through independent coding, consensus discussions, documentation of coding decisions, peer debriefing, and external qualitative expert review [21].

Recurrent conceptual patterns began to appear after approximately 15–16 written reflection forms. However, all 26 valid forms were systematically analyzed to ensure interpretive depth and to avoid overlooking subtle variations or divergent negative cases. Sample adequacy was considered using the concept of information power [22], as the study had a specific aim, a relatively homogeneous participant group, and a clearly defined course-based activity. No formal saturation grid was used. The Max Qualitative Data Analysis (MAXQDA) Analytics Pro 2024 program (MAXQDA 2024) was used as a supporting software tool for data analysis. Direct quotations from participant statements were used in the analysis process; member checking was performed to increase the validity of the themes.

### Ethical approvals

Ethical approval for the study was obtained from the Ethics Committee of Konya Selcuk University (Date: 30/10/2024, Decision No: 2024-992). Data were collected in December 2024. A formal protocol registration was not performed; however, the study aim, participant group, and data collection procedures were specified in the ethics committee application before data collection. All participants were informed about the purpose, scope, voluntary nature, and confidentiality of the study. It was clearly stated that participation, non-participation, leaving the form blank, or submitting an incomplete form would not affect students’ course grades, academic evaluation, jury evaluation, bonus points, or relationship with the instructor. Students who did not participate were not given any alternative assignment or additional task.

Written informed consent was obtained from all participants prior to data collection. Students had no prior academic or institutional relationship with the primary researcher before the study semester, during which she served as their course instructor. Because the primary researcher was also the course instructor, additional precautions were taken to reduce perceived hierarchical pressure and social desirability bias. Informed consent and data collection were conducted after the end of term exhibition and after all routine course assessments and formal grading procedures had been completed. Consent forms and anonymous written reflection forms were administered and collected by a student representative and the primary instructor was not present during data collection. Jury evaluation scores were excluded from the study data and were not used in the qualitative analysis. The study was conducted in accordance with the Declaration of Helsinki.

### Rigor and reflexivity

Trustworthiness was addressed according to the criteria of credibility, transferability, dependability, and confirmability [23]. Credibility was supported through participant validation: after pre-coding and theme development, brief theme summaries were shared with selected participants for comments and corrections. No substantive corrections or disconfirmations were reported. To enhance credibility, findings were grounded directly in participant quotes and the thematic structure was evaluated through peer debriefing. To support transferability, the institutional and course context, participant characteristics, scaffolding structure of the task, and timing of data collection were described in detail. Students were informed that participation was voluntary and that participation or non-participation would not affect their course grades or academic evaluation.

To ensure consistency, the translation was reviewed by a second translator; analysis decisions were documented to create an auditable file version [24]. To rigorously mitigate this risk, the scoring results of the jury evaluations were strictly excluded from the scope of this study, ensuring that the qualitative feedback was entirely decoupled from the academic grading loop (Fig. 1). Furthermore, to eliminate hierarchical pressure, the anonymous, identity-free written reflection forms were administered and collected solely by a student representative at the very end of the exhibition, after all routine course assessments and formal grading processes were officially finalized and locked. The primary instructor was entirely absent from the classroom during this process. This multi-layered approach was supplemented by maintaining reflexive journals and incorporating external qualitative research expertise [25, 26], ensuring the credibility and confirmability of the analysis.

## Results

All participants were female third-year midwifery students enrolled in the “Normal Birth and Postpartum Period” course. The ages of the 26 students who participated in the study ranged from 20 to 23, with a mean age of 21.2 years (SD = 0.9). The vast majority of students (88.5%; *n* = 23) stated that they had chosen the midwifery department voluntarily and that all of them enjoyed the “Normal Birth and Postpartum Period” course. It was determined that the majority (88.5%) had no experience in SGLM (Table 2). Three students were categorized as not satisfied with the process. Their written reflections were examined and integrated into the sub-themes related to time management, material/technical limitations, and satisfaction and continuation of the process.

**Table 2:** Descriptive and experiential characteristics of students (*n* = 26)

Characteristic	Number (*n*)	Percentage (%)
Gender		
Female	26	100.0
Male	0	0.0
Voluntarily chose the department		
Yes	23	88.5
No	3	11.5
Enjoyed the Normal Birth and Postpartum Period course		
Yes	26	100,0
Previous experience preparing materials		
Yes	3	11.5
No	23	88.5
Willingness to prepare materials for another course		
Yes	16	61.5
No	10	38.5
Satisfaction with the material preparation process		
Satisfied	23	88.5
Not Satisfied	3	11.5

Thematic analysis of the students’ responses to questions regarding the SGLM process within the scope of the study revealed 4 main themes and 13 sub-themes. The emerging themes provide rich descriptions of the process (Table 3).

**Table 3:** Themes and sub-themes emerging from students’ experiences in the process of SGLM

Main Theme	Sub-Themes	*n*	%
1. Individual Learning and Academic Development	1.1. Increase and retention of knowledge 1.2. Contribution to visual learning 1.3. Recognizing one’s own learning journey	23/26 9/26 9/26	88.5 34.6 34.6
2. Emotional and Individual Experience	2.1. Enjoyment and pleasure 2.2. Stress and tension 2.3. Development of self-confidence	21/26 13/26 12/26	80.8 50.0 46.2
3. Difficulties and Obstacles	3.1. Time management issues 3.2. Material and technical limitations 3.3. Indecision regarding subject and material selection	9/26 9/26 15/26	34.6 34.6 57.7
4. Instructional Direction and Application experience	4.1. Perception of applicability 4.2. Educational role experience 4.3. Clinical/field use idea 4.4. Satisfaction and continuation of the process	26/26 10/26 7/26 21/26	100.0 38.5 26.9 80.8

*n* indicates the number of students whose written reflections included the relevant sub-theme

### Individual learning and academic development

#### Increase and retention of knowledge

Most students stated that they had increased their knowledge of the course topics while preparing the material and that this knowledge had become permanent (*n* = 23).

*" I learned details that I had previously overlooked because I had to research the subject in depth. I realized that learning by creating is much more lasting than just reading. (Student 26)*

*“When selecting materials*,* I inevitably glanced over all the topics*,* which helped me remember them. It was like reviewing before an exam. I also learned more detailed information while creating my own material.” (Student 2)*.

#### Contribution to visual learning

Students used statements indicating that this process contributed to their visual learning (n = 9).

*“When preparing the material*,* I didn’t just write down what I learned; I also tried to explain it visually. This helped me internalize the information. It was the first time I thought about a topic in such depth.” (Student 18)*.

*“Because I created a visual illustration in my material about the bones*,* sutures*,* and fuses of the fetal head*,* it was much easier for me to understand and explain.” (Student 1)*.

#### Recognizing one’s own learning journey

Participants reported recognizing their own learning paths during the process (n = 9).

*“I felt like I had found a space where I could express myself. Not every student understands the lesson in the same way*,* so preparing material in my own style was very liberating.” (Student 19)*.

*“At first*,* I couldn’t make sense of it*,* but now I see how much this process has contributed to my learning. I think such hands-on activities should be included in other courses as well.” (Student 26)*.

### Emotional and personal experience

#### Enjoyment and pleasure

Many participants found the material preparation process enjoyable and fun (*n* = 21).

*“I both learned and enjoyed doing it.” (Student 25).*

*“It was nice to use my creativity. This process was fun for me; it was the first time a lesson was so enjoyable.” (Student 16)*.

#### Stress and tension

Some participants stated that they struggled and felt tense at the beginning because they did not know what to do (n = 13).

*“At first*,* I didn’t know what to do*,* I was stressed*,* but it was an event with a good outcome.” (Student 10)*.

*“It was very stressful*,* I tried hard to make it perfect.” (Student 17)*.

#### Development of self-confidence

At the end of the process, some students reported an increase in their self-confidence. This process also enabled students to experience the role of “teacher” and increased their professional confidence (n = 12).

*“I usually get very nervous when giving presentations*,* but during this process*,* I felt more comfortable explaining my material. Because I had prepared it myself and knew every step. This gave me confidence.” (Student 21)*.

*“Thanks to this process*,* I thought that I could prepare similar materials not only for class but also when sharing information as a future midwife. My confidence in myself increased.” (Student 26)*.

### Difficulties and obstacles

#### Time management issues

Since the material preparation process covered both the internship and exam periods, some students reported having difficulty managing their time. Time pressure was also noted by students who reported limited satisfaction with the activity (n = 9).

*“I struggled when exams and internships overlapped; I worked late into the night.” (Student 8)*.

*“Time management was difficult because it was challenging to keep up with other courses.” (Student 26)*.

#### Material and technical limitations

During the process, some students indicated that they had difficulty accessing sufficient materials. Some students with limited satisfaction also referred to difficulties in finding suitable materials and producing the planned model (n = 9).

*“Some materials were difficult to access*,* so I tried to do it with the resources I had.” (Student 13)*.

*“I struggled to find the main material for my work. I had to erase the fetuses I drew on my material and redraw them over and over again.” (Student 12)*.

#### Indecision regarding subject and material selection

Some students reported experiencing indecision when choosing a subject and material (*n* = 15).

*“I had trouble choosing a subject. I wanted it to be different from others.” (Student 6).*

*“I was undecided about what to do. The only difficulty was choosing the subject of the material.” (Student 4)*.

### Instructional direction and application experience

#### Perceived applicability

All students believe that the material they prepared is usable (n = 26).

*“It is very explanatory and understandable. I think that when a mother from the public or a midwifery student comes and looks at my material*,* they will find answers to all their questions about the subject.” (Student 6)*.

*“It was prepared based on scientific sources and with a simple and understandable narrative language. The fact that it is supported by visuals also appeals to different learning styles.” (Student 22)*.

#### Educational role experience

Some students stated that they felt like teachers, especially when presenting to the jury and other participants during the exhibition (n = 10).

*“This process made me feel like a teacher*,* not just a learner. The idea that the material I prepared could be a resource for other students motivated me.” (Student 4)*.

*“It made a nice and positive contribution. We worked to explain the topic to those who came* to ask questions about our material.” (Student 12).

#### Clinical/field use idea

Some students expressed the view that the materials could also be used in clinical practice (n = 7).

*“Breastfeeding counseling is taught using a single type of nipple*,* but not every client has the same nipple shape. I think my material could solve this problem.” (Student 4)*.

*“I think it would be useful not only in childbirth classes but also in pregnancy and breastfeeding classes.” (Student 13).*

#### Satisfaction and continuation of the process

Many students stated that they were satisfied with the material development process and wanted similar active learning practices to continue. In contrast, three students reported dissatisfaction or limited satisfaction with the process, mainly due to time pressure and material-related difficulties (n = 21).

*“There should be these types of practices. Not just exams*,* things like this are more educational.” (Student 19)*.

*“It was very educational. I also think it was useful in terms of showing the importance of our department and profession within the university. I thank our instructor for their hard work.” (Student 13)*.

*“I was not fully satisfied because the process was more difficult than I expected.” (Student 8)*.

## Discussion

This study explored midwifery students’ written reflections on the process of SGLM within the “Normal Birth and Postpartum Period” course. The qualitative findings showed that students generally perceived this process as a meaningful educational experience that supported their engagement with course content, self-confidence, motivation, and professional awareness. Students also reported that preparing materials helped them revisit theoretical knowledge and transform abstract concepts into visual, tactile, or teachable forms. However, these findings reflect students’ self-reported perceptions and should not be interpreted as evidence of improved learning outcomes, knowledge retention, clinical competence, or pedagogical effectiveness. Within this cautious interpretation, the findings are consistent with experiential and active learning perspectives, as well as the broader call in health professions education for learner-centered and context-sensitive educational approaches [27].

Students reported that the material preparation process encouraged them to research their topics in greater depth and helped them reinforce theoretical knowledge through an experiential activity. This finding can be interpreted in relation to Kolb’s experiential learning theory, in which learning involves the transformation of experience into knowledge [12]. In the present study, preparing materials represented a concrete experience, written reflections supported reflective observation, transforming theoretical knowledge into visual or tactile materials reflected abstract conceptualization, and presenting or applying the materials within the course context represented active experimentation. Similar to this interpretation, previous studies in nursing and health professions education have suggested that experiential learning activities may support students’ engagement with knowledge and perceived integration of theory and practice [28]. In obstetrics and gynecology education, Gadre (2019) reported that handmade models may support students’ retention of knowledge compared with video-based learning, although this evidence should be interpreted cautiously due to methodological limitations such as small sample size, non-randomized design, and author-developed outcome measures [17]. Unlike comparative studies, the present study did not assess objective knowledge retention or compare SGLM with other teaching methods; therefore, the findings should be interpreted as students’ perceived learning experiences.

Students reported that the visual characteristics of the materials helped them understand and organize course content more easily. In line with previous literature emphasizing the role of visual representation in learning, students stated that producing a visual output made abstract or complex topics more concrete and memorable [29]. Since the present study did not objectively assess cognitive restructuring or knowledge retention, these findings should be interpreted as students’ perceived learning experiences.

In the study, some students reported feeling liberated during the material preparation process and described the activity as helping them recognize their own learning journey. Allowing students freedom in both subject and method selection appeared to support choices aligned with their individual learning styles, as reflected in the generation of different material formats for the same subject matter. The literature emphasizes that autonomy and individual preferences in educational settings significantly increase learning motivation [11, 30, 31]. Consistent with this evidence, our qualitative findings suggest that providing structured flexibility within the curriculum may support students’ perceived learning motivation and professional engagement.

In the study, many students reported that the SGLM process was enjoyable, made the course more engaging, and supported their motivation toward the course. Students also stated that gaining command of the subject and presenting their materials contributed to their perceived self-confidence and professional motivation. Similarly, previous studies evaluating active learning methods have reported positive student perceptions regarding motivation, satisfaction, and course enjoyment [16, 32–34].

On the other hand, some students reported stress, tension, or anxiety during the material preparation process. Since many students had no prior experience with this type of active learning activity, the unfamiliarity and uncertainty of the task may have contributed to these feelings. The psychology literature consistently emphasizes that situations of structural uncertainty and unfamiliarity are common sources of perceived stress [35, 36]. From the perspective of cognitive load theory, tasks requiring students to understand content, design representations, produce materials, and prepare explanations simultaneously may place additional demands on working memory [18]. Therefore, SGLM activities should be supported with clear instructions, realistic time planning, and formative guidance.

In the study, students reported difficulties related to time management, material access, technical skills, and the design phase of the task. In midwifery education, where practical training runs alongside theoretical coursework, preparing a learning material may increase students’ perceived workload. These findings are consistent with previous studies reporting academic workload, clinical training demands, and time-related stressors among health professions students [37, 38]. Furthermore, from the perspective of visible learning, such student-centered tasks require appropriate instructional design, feedback, and guidance so that learning expectations and progress become clear to students [9]. Beyond student-related challenges, SGLM activities also have implementation implications for educators.

Students perceived that materials developed on topics such as preparation for childbirth, the birth process, and the postpartum period could be useful for both their own learning and future educational interactions with pregnant women, new mothers, and family members. In this respect, the material development process appeared to support students’ awareness of their educational responsibilities and professional identity. SGLM activities are also related to the learning-by-teaching and preparing-to-teach literature. Previous studies suggest that preparing instructional explanations for others may support the organization of knowledge, elaboration, and engagement with content [39–42]. However, the present study did not measure objective learning outcomes; therefore, this connection should be interpreted in terms of students’ perceived experiences. Some students also reported that preparing and presenting their materials during the exhibition helped them experience an educator role and reflect on their communication and presentation skills. These findings are consistent with literature emphasizing professional identity development, communication, and educational roles in health professions and midwifery education [34, 43–45].

Students described the process as a creative and, in some cases, art-based learning experience, and several students stated that similar practices could be supported in midwifery education. The use of methods such as knitting, embroidery, painting, ceramics, and handmade modeling illustrated how students represented course content through diverse visual and tactile formats. This finding is consistent with previous work by Advolodkina and Chahine (2017), who used felt and Velcro pieces to teach pelvic anatomy in three dimensions [16]. Similarly, creative and handmade models have been discussed as tools that may help learners visualize complex anatomical or physiological concepts and engage more actively with course content [11, 46, 47]. However, the present study did not objectively assess learning outcomes, knowledge retention, or psychomotor skill development. Therefore, the findings should be interpreted as students’ perceived experiences of transforming theoretical knowledge into educational materials rather than as evidence that the activity directly improved learning or ensured achievement of course objectives. Within this scope, students’ desire to continue SGLM in other courses may reflect the perceived motivational value of experiential and creative learning activities [32].

### Implications for future research

The findings suggest that SGLM may be perceived by students as supporting engagement, self-confidence, curiosity, and professional awareness, which is consistent with previous literature on active and experiential learning in health professions education [32, 45, 48]. However, this study did not objectively measure learning outcomes, knowledge retention, self-efficacy, practical skills, clinical competence, or cost-effectiveness. Therefore, the findings should be interpreted as students’ self-reported perceptions rather than evidence of demonstrated educational effectiveness.

Future studies should use comparative, longitudinal, and mixed-methods designs to examine the educational value of SGLM activities more comprehensively. Objective learning measures, validated motivation and self-efficacy scales, practical or clinical skill assessments, follow-up evaluations, and cost-related analyses are needed before claims can be made regarding learning outcomes, skills transfer, or cost-effectiveness [17, 45, 48]. Studies conducted in different courses, institutions, and student groups would also help clarify transferability. In addition, future research should examine the role of instructional design, scaffolding, formative feedback, educator preparedness, and institutional support in the sustainable integration of active learning approaches into midwifery and nursing education [45, 46].

A practical implication is that SGLM activities may allow students to create hands-on learning materials using accessible and low-cost resources, which may be useful in educational settings where commercial simulators are limited. However, no formal cost-effectiveness analysis was conducted. Implementation feasibility should also be considered, as SGLM activities may increase faculty workload through material review, feedback, exhibition organization, jury evaluation, and rubric-based assessment. Therefore, larger-scale applications may require structured timelines, group work, simplified rubrics, and institutional support.

### Limitations

This research was conducted with third-year students from a single foundation university’s midwifery department in southern Türkiye, which limits transferability to other institutional, regional, and educational contexts. Because foundation universities serve distinct socioeconomic demographics and provide different levels of resource accessibility compared to public universities, the transferability of these results should be interpreted with caution. As the study used a qualitative design, the findings reflect students’ subjective experiences.

Furthermore, participants’ voluntary choice of the midwifery department, high course satisfaction, and very high course enjoyment ratings may have contributed to positive response or social desirability bias, particularly given the instructor–researcher relationship and the highly motivated participant profile. Therefore, these findings should be interpreted cautiously as self-reported perceptions rather than as evidence that the activity was uniformly beneficial for all students. Selection bias is also possible because 13 of the 39 eligible students did not provide usable written reflection data and their experiences may have differed systematically from those of the participants.

Despite collecting anonymous forms after grading was completed and in the absence of the instructor, the primary researcher’s dual role as course instructor may still have introduced a perceived power imbalance and social desirability bias that could not be fully eliminated. Another limitation is that data were collected through written reflections rather than interviews or focus groups, which limited probing and follow-up questioning. Although the responses of students who reported dissatisfaction or limited satisfaction were reviewed separately and incorporated into the relevant sub-themes, the written reflection format limited deeper exploration of less favorable experiences. Although the responses of students who reported dissatisfaction or limited satisfaction were reviewed separately and incorporated into the relevant sub-themes, the written reflection format limited deeper exploration of these experiences. Therefore, the findings should be interpreted as a thematic analysis of written reflections.

Lastly, since this study relied purely on cross-sectional qualitative feedback collected after a single course-based activity and did not include baseline measurements, validated quantitative instruments, or a comparative control group, causal inferences cannot be made. Students’ reported experiences may have been influenced by maturation during the semester, other course experiences, the wording of the reflection questions, or pre-existing motivation and confidence. No follow-up assessment was conducted; therefore, the study cannot address long-term retention, clinical application, or sustained effects of the SGLM activity. Accordingly, the findings should be interpreted as exploratory self-reported perceptions rather than objective educational outcomes or demonstrated pedagogical effectiveness.

## Conclusion

This study explored midwifery students’ written reflections on the process of SGLM within a single course-based educational context. The findings suggest that students perceived the process as supporting their engagement with course content, motivation, self-confidence, professional awareness, and experience of an educational role. At the same time, students also reported challenges related to stress, time management, material access, technical skills, and uncertainty during preparation. Therefore, SGLM activities should be planned with clear guidance, realistic time allocation, formative feedback, and adequate instructional support. Overall, carefully structured experiential learning activities such as SGLM may be considered as a supportive strategy alongside theoretical instruction in midwifery education.

## Supplementary Information

Supplementary Material 1.

Supplementary Material 2.

Supplementary Material 3.

## Data Availability

The anonymized qualitative data generated and analyzed during the current study are not publicly available because the written reflections may contain context-specific expressions that could potentially compromise participant confidentiality. However, de-identified excerpts or relevant parts of the dataset may be made available from the corresponding author upon reasonable request, subject to ethical approval, and confidentiality considerations.
