# Supplementary material for: Student-generated learning materials as active learning: a qualitative study of midwifery students’ experiences
Source: BMC Med Educ. 2026 Jul 1;26:1504. doi: 10.1186/s12909-026-09749-9 (PMC13595507; doi:10.1186/s12909-026-09749-9)
Supplement: Supplementary file 1 [file 12909_2026_9749_MOESM1_ESM.docx]

**Supplementary Figure 1**. Examples of SGLM prepared by midwifery students within the scope of the course

**Note:** This supplementary figure includes only the educational materials prepared by students who granted permission for visual presentation.
