# Supplementary material for: Student-generated learning materials as active learning: a qualitative study of midwifery students’ experiences
Source: BMC Med Educ. 2026 Jul 1;26:1504. doi: 10.1186/s12909-026-09749-9 (PMC13595507; doi:10.1186/s12909-026-09749-9)
Supplement: Supplementary file 2 [file 12909_2026_9749_MOESM2_ESM.docx]

| **Material type** | **Examples** | **n (%)** |
| --- | --- | --- |
| Hands-on model /model-type materials | Breast model, placenta model, pelvic bones, fetal head, uterus, birth stages model | 22 (84.62%) |
| Artistic/handmade materials | Painting, handicraft, knitting, decorative educational object | 3 (11.54%) |
| Poster/visual educational materials | Poster, infographic, visual explanation board | 1 (3.84%) |
| Digital content | Digital presentation, video, digital visual content | 0 (00.0%) |
| Other | Materials not included in the above categories | 0 (00.0%) |

**Supplementary Table 2.** Distribution of SGLM types
