# Supplementary material for: Student-generated learning materials as active learning: a qualitative study of midwifery students’ experiences
Source: BMC Med Educ. 2026 Jul 1;26:1504. doi: 10.1186/s12909-026-09749-9 (PMC13595507; doi:10.1186/s12909-026-09749-9)
Supplement: Supplementary file 3 [file 12909_2026_9749_MOESM3_ESM.docx]

**Consolidated criteria for reporting qualitative studies (COREQ): 32-item checklist for manuscript in title:** **“Student-generated learning materials as active learning: a qualitative study of midwifery students’ experiences”**

| **No** | **Item** | **Guide questions/description** | **Response** |
| --- | --- | --- | --- |
| **Domain 1: Research team and reflexivity** | | |  |
| Personal Characteristics | | |  |
| 1. | Interviewer/facilitator | Which author/s conducted the interview or focus group? | Written reflection data were collected using an anonymous structured form. The process was administered without the presence of the primary instructor. |
| 2. | Credentials | What were the researcher's credentials? *E.g. PhD, MD* | Researcher credentials: PhD, |
| 3. | Occupation | What was their occupation at the time of the study? | Assistant Professor at the time of the study. |
| 4. | Gender | Was the researcher male or female? | Female |
| 5. | Experience and training | what experience or training did the researcher have? | qualitative research |
| Relationship with participants | | |  |
| 6. | Relationship established | Was a relationship established prior to study commencement? | The primary researcher was also the course instructor and had an instructor–student relationship with the participants during the semester. Prior to this specific semester, the students had no previous academic acquaintance or institutional relationship with the primary researcher. This dual role was acknowledged as a potential source of perceived power imbalance and social desirability bias. |
| 7. | Participant knowledge of the interviewer | What did the participants know about the researcher? e*.g. personal goals, reasons for doing the research* | Students knew the researcher as their course instructor for the first time that semester. Before data collection, students were informed about the study aim, voluntary participation, confidentiality procedures, and that participation or non-participation would not affect their course grades or academic evaluation. |
| 8. | Interviewer characteristics | What characteristics were reported about the interviewer/facilitator? e.g. *Bias, assumptions, reasons and interests in the research topic* | The primary researcher was a midwifery academic and the instructor of the course. The researcher was aware that this dual role could influence students’ responses. Reflexive notes, peer debriefing, and external qualitative expert review were used to support credibility and reduce interpretive bias. |
| **Domain 2: study design** | | |  |
| Theoretical framework | | |  |
| 9. | Methodological orientation and Theory | What methodological orientation was stated to underpin the study? *e.g. grounded theory, discourse analysis, ethnography, phenomenology, content analysis* | This study was designed as an exploratory qualitative descriptive study using reflexive thematic analysis. |
| Participant selection | | |  |
| 10. | Sampling | How were participants selected? *e.g. purposive, convenience, consecutive, snowball* | Purposive sampling from students who prepared materials. |
| 11. | Method of approach | How were participants approached? e*.g. face-to-face, telephone, mail, email* | Students were invited to participate during the course period and were informed that participation was voluntary. |
| 12. | Sample size | How many participants were in the study? | 26 students. It is mentioned in the sample section |
| 13. | Non-participation | How many people refused to participate or dropped out? Reasons? | Thirteen eligible students were classified as non-participants because they did not provide usable written reflection data. No student explicitly refused participation or formally withdrew after invitation. |
| Setting | | |  |
| 14. | Setting of data collection | Where was the data collected? e*.g. home, clinic, workplace* | Data were collected in the course/classroom setting at the end of the semester, after the end of term exhibition and after all routine course assessments and formal grading procedures had been completed. The primary instructor was not present during data collection. |
| 15. | Presence of non-participants | Was anyone else present besides the participants and researchers? | A student representative administered and collected the anonymous written reflection forms. The primary instructor was not present during this process. |
| 16. | Description of sample | What are the important characteristics of the sample? *e.g. demographic data, date* | Third-year midwifery students; demographic details in Table 2. |
| Data collection | | |  |
| 17. | Interview guide | Were questions, prompts, guides provided by the authors? Was it pilot tested? | Data were collected using a structured written reflection form consisting of six open-ended questions developed based on the literature and expert opinion. No oral interviews or focus groups were conducted. |
| 18. | Repeat interviews | Were repeat interviews carried out? If yes, how many? | No |
| 19. | Audio/visual recording | Did the research use audio or visual recording to collect the data? | Not Applicable |
| 20. | Field notes | Were field notes made during and/or after the interview or focus group? | Reflexive notes were kept by the researcher during the research and analysis process to document methodological decisions, assumptions, and potential bias related to the instructor–researcher dual role. |
| 21. | Duration | What was the duration of the interviews or focus group? | Written responses took approximately 20–30 minutes. |
| 22. | Data saturation | Was data saturation discussed? | Recurrent conceptual patterns began to appear after approximately 15–16 written reflection forms; however, all 26 valid forms were systematically analyzed to ensure interpretive depth and to avoid overlooking subtle variations or divergent negative cases. No formal saturation grid was used. |
| 23. | Transcripts returned | Were transcripts returned to participants for comment and/or correction? | Not applicable in the sense of interview transcripts. However, brief summaries of preliminary themes were shared with selected participants for participant validation, and they were invited to provide comments or corrections. |
| **Domain 3: analysis and findings** | | | |
| Data analysis | | | |
| 24. | Number of data coders | How many data coders coded the data? | Two coders analyzed the data. |
| 25. | Description of the coding tree | Did authors provide a description of the coding tree? | Coding tree with themes and subthemes provided in Table 3. |
| 26. | Derivation of themes | Were themes identified in advance or derived from the data? | Themes were derived inductively from the data. |
| 27. | Software | What software, if applicable, was used to manage the data? | Software: MAXQDA was used for data management. |
| 28. | Participant checking | Did participants provide feedback on the findings? | Brief summaries of preliminary themes were shared with six students for participant validation; no substantive corrections or disconfirmations were reported. |
| **Reporting** | | | |
| 29. | Quotations presented | Were participant quotations presented to illustrate the themes / findings? Was each quotation identified? e*.g. participant number* | Quotations with participant codes are presented in the Results. |
| 30. | Data and findings consistent | Was there consistency between the data presented and the findings? | Yes. |
| 31. | Clarity of major themes | Were major themes clearly presented in the findings? | Yes. |
| 32. | Clarity of minor themes | Is there a description of diverse cases or discussion of minor themes? | Minor themes and diverse cases were also discussed. |
